# Model Selection Approach Suggests Causal Association between 25-Hydroxyvitamin D and Colorectal Cancer

**DOI:** 10.1371/journal.pone.0063475

**Published:** 2013-05-24

**Authors:** Lina Zgaga, Felix Agakov, Evropi Theodoratou, Susan M. Farrington, Albert Tenesa, Malcolm G. Dunlop, Paul McKeigue, Harry Campbell

**Affiliations:** 1 Centre for Population Health Sciences, University of Edinburgh, Edinburgh, United Kingdom; 2 Colon Cancer Genetics Group and Academic Coloproctology, Institute of Genetics and Molecular Medicine, University of Edinburgh, Western General Hospital, Edinburgh, United Kingdom; 3 Andrija Stampar School of Public Health, Medical School, University of Zagreb, Zagreb, Croatia; 4 Pharmatics Limited, Edinburgh, United Kingdom; 5 The Roslin Institute, Royal (Dick) School of Veterinary Studies, University of Edinburgh, Midlothian, United Kingdom; IFOM, Fondazione Istituto FIRC di Oncologia Molecolare, Italy

## Abstract

**Introduction:**

Vitamin D deficiency has been associated with increased risk of colorectal cancer (CRC), but causal relationship has not yet been confirmed. We investigate the direction of causation between vitamin D and CRC by extending the conventional approaches to allow pleiotropic relationships and by explicitly modelling unmeasured confounders.

**Methods:**

Plasma 25-hydroxyvitamin D (25-OHD), genetic variants associated with 25-OHD and CRC, and other relevant information was available for 2645 individuals (1057 CRC cases and 1588 controls) and included in the model. We investigate whether 25-OHD is likely to be causally associated with CRC, or vice versa, by selecting the best modelling hypothesis according to Bayesian predictive scores. We examine consistency for a range of prior assumptions.

**Results:**

Model comparison showed preference for the causal association between low 25-OHD and CRC over the reverse causal hypothesis. This was confirmed for posterior mean deviances obtained for both models (11.5 natural log units in favour of the causal model), and also for deviance information criteria (DIC) computed for a range of prior distributions. Overall, models ignoring hidden confounding or pleiotropy had significantly poorer DIC scores.

**Conclusion:**

Results suggest causal association between 25-OHD and colorectal cancer, and support the need for randomised clinical trials for further confirmations.

## Introduction

In 1980, it was first hypothesised that vitamin D is a protective factor against colorectal cancer (CRC) [1]. It has subsequently been shown that higher vitamin D intake [2], higher serum 25-hydroxyvitamin D (25-OHD) [3] and residence in regions with strong UVB radiation [4] are all associated with lower CRC risk and cancer death [5]. The majority of the available evidence comes from ecologic correlations or observational studies. Unbiased attempts to investigate causality in these studies are unreliable, as study design cannot completely account for the effect of potential confounders such as obesity or physical activity [6], [7]. Nevertheless, experimental studies [8], [9], randomized controlled trials [2], [10] and application of Hill’s criteria for causality [11], [12] support a possible causal role of vitamin D deficiency in colorectal cancer. The volume of observational and *in vitro* evidence and the potential large public health importance should associations prove to be causal, require further investigation.

While awaiting results from randomised clinical trials, statistical and machine learning methods allow the investigation of causality in observational studies. One such method is Mendelian randomization (MR). MR is an application of instrumental variable (IV) analysis that uses genetic polymorphisms as instruments [13], [14], [15]. It has become increasingly more popular, since genome-wide association studies (GWAS) identified numerous genetic variants that can be used as “instruments” [16].

Conventional MR approaches assume that: (i) genotypes are randomized; (ii) genetic variants considered as instruments affect the outcome *only* by modifying the biomarker, i.e. there are no pleiotropic effects of these variants on the outcome; (iii) variations between true and observed biomarkers are negligible (no observation noise) [17], [18], [19], [20]. If these assumptions hold, inference of causality from observational data is theoretically valid, although conclusions are sensitive to the chosen instruments [21] and may not be valid when the effects of the instruments on the biomarkers are weak. Despite their popularity, it has been argued that MR methods push the problem of causal inference to another realm, as their assumptions are generally unverifiable [22]. For example, it is rarely possible to exclude pleiotropy or estimate effects of such exclusions on the resulting estimate, especially for multiple instruments [23], [24]. Also, in classic MR it is difficult to assess how the causal estimates are affected by different assumptions about distributions of the latent confounders.

Another important limitation of MR is that it lacks a formal model comparison framework for inferring the *direction* of causality when pleiotropy and confounding cannot be excluded as possible explanations. The classic approach estimates the size of the causal effect [25], but does not assess the relative value of causal *vs.* reverse causal explanations. This may not matter in a long-term cohort study where the temporal sequence from biomarker to outcome is clear, but it limits the ability to infer causality from cross-sectional or case-control data. Also note that in pleiotropic models the causal and reverse models are not nested, and classical tests for nested cannot be easily used. A more general approach to learning the direction of causality is the Likelihood-based Causality Model Selection (LCMS) method suggested by [26], who propose selection of the best modelling hypothesis by comparing likelihood-based scores for direct causal, reverse, and pleiotropic models. While this approach relaxes the assumption of no pleiotropy of the classic MR method, it does not allow for latent confounders or measurement noise. Additionally, because their method is not Bayesian, it cannot be easily scaled to large problems where high-dimensional genotypes and/or phenotypes are used as instruments.

We have previously performed a MR analysis to investigate the possible causal effect of plasma 25-OHD on colorectal cancer risk [27]. Our results were inconclusive and a causal relationship between low 25-OHD and CRC was neither clearly demonstrated nor excluded.

In this study, we set out to investigate the causal effect of 25-OHD on colorectal cancer risk. We extend conventional approaches (MR and LCMS) by: (i) allowing pleiotropic links between the instruments and disease, (ii) accounting for the noise in the measurement and (iii) modelling of “hidden confounders”, i.e. unmeasured factors that can affect biomarker and disease. We proceed by selecting the best modelling hypothesis according to Bayesian predictive scores, and investigate its consistency for a broad range of prior assumptions. Our approach builds on the strengths of MR and LCMS but relaxes their restrictive assumptions, which results in models that better fit the data according to the considered criteria.

## Methods

We studied a subset of individuals from the SOCCS Study (1999–2006) [27], [28]. In total, 2645 individuals with all relevant measurements available were included in this study (1057 cases and 1588 controls). Ethical approval for the SOCCS study was obtained from the MultiCentre Research Ethics committee for Scotland (reference number 01/0/05) and from the Research and Development Office of NHS Lothian (reference number 2003/W/GEN/05). All participants gave informed written consent. The subjects completed a questionnaire enquiring about lifestyle. Questionnaire collected data on general medical history, physical activity (hours of cycling and other sports activities, 4 groups), socio-economic status (Carstairs Deprivation Index), smoking habits, regular intake of aspirin and NSAIDs, height, weight, and other. Participants also completed a semi-quantitative food frequency (http://www.foodfrequency.org) and supplements questionnaires, which were used to calculate the vitamin D intake (see [27], [29]).

Total plasma 25-OHD (25-OHD_2_ and 25-OHD_3_), the main storage form of vitamin D, was measured by liquid chromatography-tandem mass spectrometry (LC-MS/MS) method [30]. 25-OHD concentration was standardized to remove the prominent effect of the season when blood was taken, and May-adjusted measurement was used in the analyses (as described in [29]).

In this study, we used 16 SNPs associated with CRC in GWAS: rs6691170, rs6687758, rs10936599, rs16892766, rs7014346, rs10795668, rs3802842, rs7136702, rs11169552, rs4444235, rs4779584, rs9929218, rs4939827, rs10411210, rs961253, rs4925386 [31], [32], [33], [34], [35], [36] and four SNPs associated with 25-OHD: rs2282679, rs12785878, rs10741657, rs6013897 [37]. We have reduced dimensionality of genetic factors to 6 principal components.

### Probabilistic Graphical Modelling

Relationships between biomarkers and outcomes can be described by “Bayesian networks” represented by directed acyclic graphs, where **nodes** correspond to random variables, **edges** describe conditional independence structures, and every two nodes are conditionally independent of each other given their parents. Such models have been widely explored in statistical and machine learning literature; their key advantage is that they can sometimes be used to differentiate causality from mere statistical associations [38], [39], [40].

As argued e.g. by MacKay (35.3, [41]), a Bayesian approach to causality inference may be based on *model selection*, where models describing different causal hypotheses are considered and compared. For example, when priors on confounding and pleiotropic effects are specified, the weight of evidence favouring a causal model over an alternative one can be evaluated even though the classical criteria for identification of causal effects in graphical models [42], [43] are not met. The fact that the same model is selected for a broad range of domain-specific priors may indicate the direction of causality (which may need to be further validated through controlled experiments). This approach is attractive, because it is applicable in real-world situations where both confounding and pleiotropy may be present.

**Figure 1:**
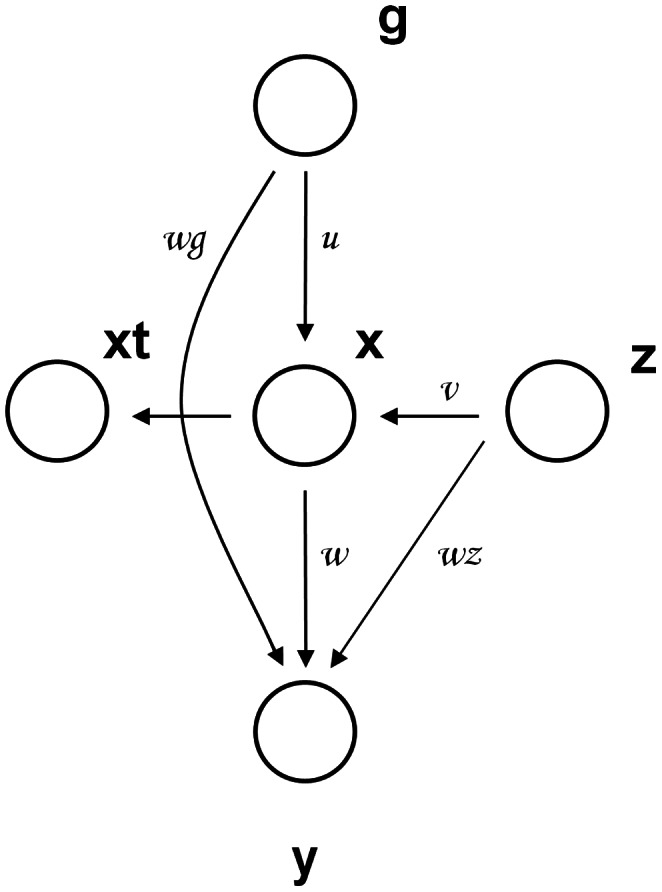
Graphical representation of the basic model: *y* – outcome (colorectal cancer, CRC); *x* – true concentration of the biomarker, 25-OHD; *xt* – measured concentration of the biomarker, 25-OHD; *g* – a vector of predictor variables: age, sex, smoking, BMI, physical activity, family history, NSAIDs intake, socioeconomic status, total caloric intake, alcohol intake, consumption of red meat, dietary vitamin D intake and SNPs associated with CRC or 25-OHD; *z* – unmeasured, hidden confounders. Link *u* represents the effect of predictor variables on 25-OHD, *w* is the effect of 25-OHD on CRC, *wg* is the effect of predictor variables on the CRC, *v* is the effect of unmeasured confounders on the 25-OHD and *wz* is the effect of unmeasured confounders on colorectal cancer.

The graphical structure of the generic model considered in this paper is shown on **Figure 1**. This extends the previously introduced method of [44] by allowing for pleiotropic effects of genotypes on biomarkers and outcomes. We consider several variants of basic model, e.g. by reversing the direction of the link between vitamin D and colorectal cancer, or removing it entirely. For all such models, we compute likelihood-based scores which indicate how well the model fits the data, in accordance with recently introduced approach [45], [46], [47].

#### Outcome, Biomarker and Predictor variables

We examine the relationship between colorectal cancer (outcome, *y*) and May-standardised plasma 25-OHD (biomarker, with the true unobserved concentration denoted by *x* and the corresponding noisy measurement denoted by *xt*) as shown on **Figure 1**. Note that *xt* accommodates measurement error and biological oscillations. We account for *known* confounding by including the vector of predictor variables *g,* which contains data on general and environmental factors (age, sex, BMI, physical activity, family history of CRC, NSAIDs intake, socio-economic status, total caloric intake, alcohol intake, smoking, consumption of red meat and dietary vitamin D intake) and genetic factors. Prior to the analysis, all predictor variables were scaled to have: mean = 0 and SD = 1.

#### Unmeasured (or hidden) confounders

We assume that joint effects of unmeasured confounders on biomarker and outcome are approximately additive and may be summarized by a hidden (latent) variable *z* (**Figure 1**), where *z* follows a Gaussian distribution with mean = 0 and SD = 1. Gaussianity of latent factors is a standard assumption of mixed linear models [48] and may be justified by the Central Limit theorem (which postulates that the sum of a large number of independent effects is approximately normally distributed, under certain conditions). The constraint on the variance of the confounder is needed to ensure identifiability of its effect on biomarkers and outcomes; we choose it so that confounder *z* lies on the same scale as the scaled predictor variables. During inference the confounder is marginalized out by computing averages over its probability distribution, which is a standard way of accounting for hidden variables in probability theory [41].

#### Model parameterization

Agakov et al. introduced the Sparse Instrumental Variable method (SPIV) [46]. They assume that all conditional distributions in the model shown on **Figure 1** are linear Gaussians, with the inverse gamma priors on the variances of noise terms, and sparsity-inducing Laplace priors on coefficients of the linear mappings [46]. They consider the *maximum a posteriori* approximation of inference; define an expectation-maximization (EM) algorithm for fitting their model to data, and use cross-validation to further tune hyperparameters. We largely follow this construction, but assume a binary outcome variable *y* (case/control) and a sparse logistic regression model for the probability of CRC given the genotypes, biomarker, and hidden confounders. Also, in contrast to [46], instead of using point estimates of the parameters, we consider the more general full Bayesian treatment approximated by Markov Chain Monte Carlo (MCMC).

#### Priors/parameters

Similarly to [49] we considered zero-mean Laplace priors on the linear coefficients with the concentration hyperparameter *gam1*. Models with larger *gam1* are more likely to have their links pruned in the posterior mode (see **Figure S1**).We investigate the relationship between CRC and 25-OHD for a range of prior distributions (assuming *gam1* is 0.025 unless stated otherwise). The concentration around zero encodes our belief that large genotypic and phenotypic effects are unlikely, while the fat tails of the Laplace component allow for possible rare large associations.

We denoted precisions (inverse variances) of linear predictors as *precx*, *precxt*, *precy* and *precz* for the true 25-OHD, measurement of 25-OHD, effects on disease status, and unmeasured confounders respectively. For these, we have used both fixed values for ensuring identifiability of the random effects and indicative of our beliefs in the magnitude of the observation noise, and the conjugate Gamma priors. Smaller values of the precisions correspond to wider confidence intervals associated with every measurement.

#### Probabilistic inference and model selection

Several likelihood-based scores may in principle be considered [50], [51]. Here we select the best model by using deviance information criterion (DIC) readily computable from MCMC samples [51]. DIC balances quality of fit and complexity of a model, which helps avoid overfitting. Preferred modelling hypotheses are characterized by lower DICs, providing the best combination of quality and simplicity.

Models are compared by examining their DIC score *differences*. Roughly, absolute differences of above 10 units definitely rule out the model with the higher DIC, and differences between 5 and 10 are substantial [51], [52]. We investigate consistency of the best model under different assumptions about priors on the fixed effects of the covariates, random effects of the confounders, and the measurement noise. For the best such settings, we also compare posterior means of the models’ deviances.

### Experiments

**Figure 2:**
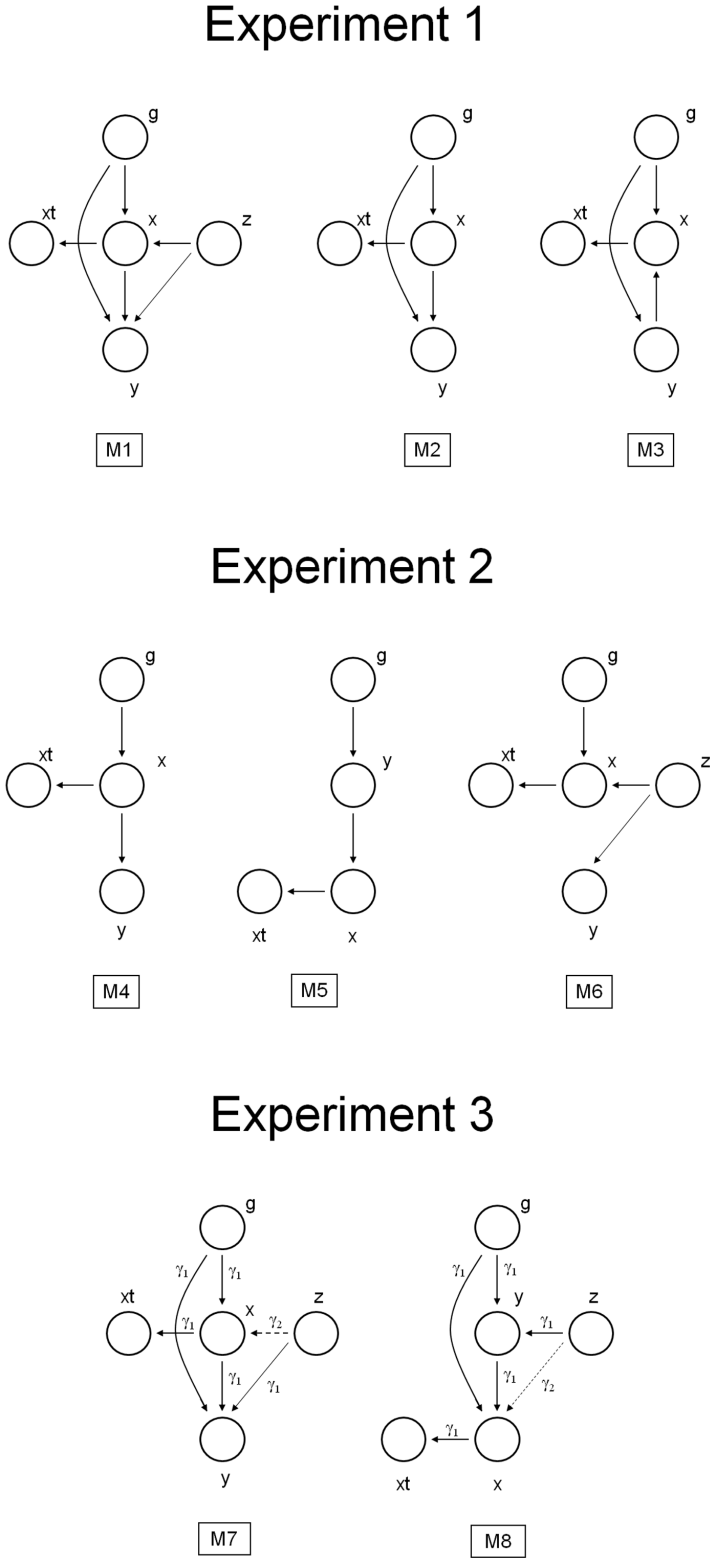
Graphical representation of models compared in Experiments 1 to 3 are shown. **A. Experiment 1.** M1 - full causal model with confounders, M2 - causal model without confounders, and M3 - reverse model without confounders. **B. Experiment 2.** We compare conventional causal (M4) and conventional reverse causal (M5) models (both (i) assume absence of pleiotropic effects of instruments on biomarkers and outcomes, (ii) explicitly exclude unmeasured confounders from modelling and (iii) account for the noise in the measurement) with the model where the association between the biomarker and outcome is modelled *entirely* by unmeasured confounders (M6). **C. Experiment 3.** We compare full causal (M7) and full reverse causal model (M8), allowing for pleiotropic relationships and accounting for hidden, unmeasured confounders.

In all experiments, we used the entire set of genotypic scores and environmental factors associated with either CRC or 25-OHD. The aim of **experiment 1** was to determine the importance of unmeasured confounders and their implication on the inference of causality. We compared 3 models: the full causal model with confounders (M1), the causal model *without* confounders (M2), and the reverse model *without* confounders (M3) (**Figure 2A**). We allowed for a possibility of pleiotropic dependencies where both the biomarker and the outcome were affected by predictor variables (the genotypes and environmental factors). The models were then compared for a range of prior distributions and assumptions about the observation noise, and the best modelling hypothesis was selected based on the DIC score.

In **experiment 2,** we considered the noisy extensions of the conventional causal (M4) and reverse (M5) models of the LCMS approach [26], [53] with a model where the association between the biomarker and outcome was explained entirely by an unmeasured confounder (M6), as shown on **Figure 2B**. The purpose of this experiment was two-fold: (i) to demonstrate restrictiveness of the assumption of no latent confounders in LCMS, and (ii) to show that a Bayesian treatment of the classic instrumental variable method [44] would not be able to identify causality by favouring a non-causal over a causal explanation. As in experiment 1, we selected the best model for a range of prior parameter settings.

The purpose of **experiment 3** was to compare the full causal and reverse models where the confounders were modelled explicitly (**Figure 2C**). Note that both of these models are likelihood-equivalent; e.g. for each setting of parameters of one there exists a setting of parameters of the other which results in an identical likelihood. The approach considered here handled such symmetry by choosing the Laplace prior distribution on the magnitudes of the linear effects, which encoded our prior belief that very large genotypic and phenotypic effects are rare (see **Appendix S1**).

In the **exploratory phase of experiment 3**, we considered independent priors on the direct associations between the biomarker and the outcome (*gam1,w* link) and the confounding effects (*gam2*, *v* and *wz* links), which were made different in order to further increase the flexibility of the method. A random sample of 500 cases and 500 controls was used to make an exploration of different prior assumptions more efficient. We performed multiple runs of the Markov chains from random initializations to account for possible variations in the deviance scores (see **Methods S1** for more details) for a broad range of prior distributions.

**In the final phase of experiment 3,** using the complete dataset we compared the full causal (M7) and reverse (M8) models where the confounders were modelled explicitly. We performed multiple repetitions keeping sparsity parameter *gam1* fixed to the best value from the earlier low-dimensional phase, but varied precisions to check consistency of the results.

## Results

The study population is described in **Table 1** **.** Both crude and May-standardised 25-OHD levels were strongly associated with CRC in the univariate model (p = 1.2E-10 and 6.9E-9, respectively), model adjusted for age and sex (p = 3.5E-10 and 2.9E-8, respectively) and in fully adjusted model (p = 5.5E-10 and 2.0E-8, respectively). Moreover, predicted vitamin D level (using all covariates) was also associated with CRC (p = 0.048), suggesting that chosen covariates are predictive of vitamin D and can indeed be considered as valid candidate instruments. Results were consistent when data was split into training and testing datasets (data not shown).

**Table 1:** Study cohort.

Variable	ALL	CONTROLS	CASES
**N**	2645	1588	1057
**Age, years**	62.8 (10.3)	62.9 (10.2)	62.6 (10.4)
**Gender, % female**	41.98	41.44	43.99
**25-OHD, ng/ml**	11.25 (6.96–16.94)	12.25 (7.60–18.00)	10.25 (5.94–15.36)
**BMI**	26.69 (4.50)	26.77 (4.67)	26.57 (4.24)
**Physical Activity, N (%)**			
cat 1	1471 (55.61)	861 (54.22)	610 (57.71)
cat 2	686 (25.54)	415 (26.13)	271 (25.64)
cat 3	309 (11.68)	197 (12.41)	112 (10.60)
cat 4	179 (6.77)	115 (7.24)	64 (6.05)
**Family health risk, N (%)**			
low	2471 (93.42)	1572 (98.99)	899 (85.05)
medium	158 (5.97)	15 (0.94)	143 (13.53)
high	16 (0.6)	1 (0.06)	15 (1.42)
**NSAIDS, N (%)**			
yes	900 (34.03)	573 (36.08)	327 (30.94)
no	1745	1015	730
**Carstairs Deprivation Index, N (%)**			
1	255 (9.64)	156 (9.82)	99 (9.37)
2	579 (21.89)	344 (21.66)	235 (22.23)
3	730 (27.6)	442 (27.83)	288 (27.25)
4	616 (23.29)	368 (23.17)	248 (23.46)
5	256 (9.68)	156 (9.82)	100 (9.46)
6	147 (5.56)	88 (5.54)	59 (5.58)
7	62 (2.34)	34 (2.14)	28 (2.65)
**Energy intake, Kcal/day**	2575 (982)	2521 (926)	2657 (1057)
**Alcohol, g/day**	7.9 (1.8–18.8)	8.1 (1.8–19.34)	7.6 (1.9–18.6)
**Smoking, N (%)**			
never	1155 (43.67)	699 (44.02)	456 (43.14)
former	1062 (40.15)	617 (38.85)	445 (42.1)
current	428 (16.18)	272 (17.13)	156 (14.76)
**Red meat, portion/day**	1.24 (0.8–1.72)	1.23 (0.79–1.75)	1.25 (0.82–1.69)
**Vitamin D (from food), µg/day**	4.27 (3.16–5.79)	4.42 (3.24–5.95)	4.04 (3.06–5.59)
**Vitamin D (from supplements), N (%)**			
>5 µg/day	151 (5.71)	85 (5.35)	66 (6.24)
>2.5 µg/day	498 (18.83)	307 (19.33)	191 (18.07)

Mean (standard deviation) or median (interquartile range) is shown for continuous variables and number (percent) is shown for categorical variables.

Physical activity is estimated from the reported hours of cycling and other sports activities (4 categories) and Carstairs Deprivation Index was used to describe socio-economic status.

### Experiment 1. Importance of Confounders for the Inference of Causality

For the first setting in Experiment 1, DIC scores for causal and reverse causal models without confounders were DIC_M2_ = 42,132 and DIC_M3_ = 41,911, respectively. The significantly lower DIC score for reverse causal model (DIC difference = 221 units) indicates its superiority over the causal model. However, DIC score for the full causal model with confounders (M1) was significantly lower (DIC_M1_ = −3,797), yielding a very large DIC difference of 45,929 and 45,708 units in support of M1, when compared to M2 and M3, respectively. Results were consistent across all tested settings (**Table 2** **).** This suggests that the model accounting for unmeasured confounders *by far* outperforms models without confounders.

**Table 2:** Likelihood-based deviance information criterion (DIC) scores for 3 models compared in Experiment 1 are shown.

MODEL	setting 1	setting 2	setting 3
full causal model with confounders	−3,797**	−2,547**	−3,003**
causal model without confounders	42,132	212,173	21,300
reverse causal model without confounders	41,911	210,996	21,183

DIC has been computed from MCMC samples; preferred modelling hypotheses are characterized by lower DICs. The full causal model with confounders (M1) suggests causal relationship between 25-OHD and colorectal cancer and also models hidden confounding, causal model without confounders (M2) also proposes causal relationship, but hidden confounding is disregarded, and reverse model without confounders (M3) proposes that colorectal cancer leads to lower levels of 25-OHD, also ignoring hidden confounding. Digits after decimal point have been omitted from the table for clarity.

Setting 1: *precxt* = 200, *precx* = 200, *precy* = 100; Setting 2: *precxt* = 1000, *precx* = 1000, *precy* = 0.1; Setting 3: *precxt* = 100, *precx* = 100, *precy* = 100. Sparsity parameter gamma is set to 0.025 in all models. In model with confounders (M1), *precz* = 1.

** indicates the best model for each setting.

### Experiment 2. Comparison with LCMS Models

DIC scores for the conventional causal and reverse causal models considered by LCMS [26], [53] were DIC_M4_ = 43,347 and DIC_M5_ = 41,915, respectively, for the first setting in Experiment 2. A DIC score difference of 1,432 in favour of M5 suggests that reverse causal relationship between 25-OHD and CRC is more likely. However, a model that assumes *only* an indirect association between 25-OHD and CRC through unmeasured confounders (M6), fits the data significantly better than either of the previous models (M4 and M5), as is suggested by DIC score differences of 43,266 and 41,834 units, respectively. Results were consistent across all tested settings (**Table 3** **).**

**Table 3:** Likelihood-based deviance information criterion (DIC) scores for conventional causal (M4) and conventional reverse causal (M5) models, both (i) assume absence of pleiotropic effects of instruments on biomarkers and outcomes, (ii) explicitly exclude unmeasured confounders from modelling and (iii) account for the noise in the measurement; and for the model where the association between the biomarker and outcome is modelled *entirely* by unmeasured confounders (M6); these models have been compared in Experiment 2.

MODEL	setting 1	setting 2	setting 3
conventional causal(without confounders)	43,347	218,230	21,883
conventional reverse(without confounders)	41,915	211,254	21,189
no causal link but accountingfor unmeasured confounders	81**	−1,549**	689**

Digits after decimal point have been omitted from the table.

Setting 1: *precxt* = 200, *precx* = 200, *precy* = 100; Setting 2: *precxt* = 1000, *precx* = 1000, *precy* = 0.1; Setting 3: *precxt* = 100, *precx* = 100, *precy* = 100. Sparsity parameter gamma is set to 0.025 in all models. In model with confounders (M6) *precz* = 1.

** indicates the best model for each setting; preferred modelling hypotheses are characterized by lower DICs.

### Experiment 3. Inference of Causality between Plasma 25-OHD and CRC

In the exploratory stage of experiment 3, we performed 30 comparisons varying *gam1* and *gam2*. Unsurprisingly, for sparser models (higher values of *gam1*) the difference in the scores of full causal and reverse models becomes less significant. This is intuitive, because for larger *gam1* the models are approximately decoupled, and any difference is largely due to the sampling noise (**Figure S1**). Mean DIC was calculated for each *gam1* setting, and it was confirmed that dense models fit the data better (−2801.12, −1816.54, −1598.58, −1571.33 and −1557.48, respectively).

**Figure 3:**
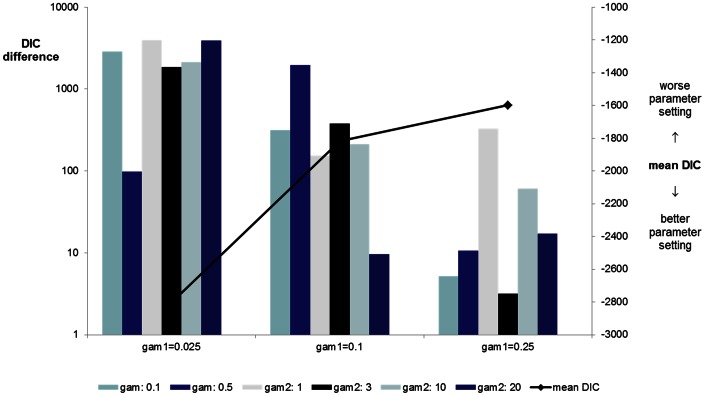
Likelihood of causal association between low 25-OHD and colorectal cancer is compared with the reverse causal hypothesis, (proposing CRC leads to lower 25-OHD), in a subset of data comprising a random sample of 500 cases and 500 controls. DIC score differences arising from the comparison of the full causal and reverse causal models, for a range of parameter settings are shown. Positive values indicate preference for the causal model. Mean DIC (black line) represents the average DIC for all causal and reverse causal models considered (lower mean DIC scores suggest better models), for any given setting of sparsity *gam1* parameter (higher *gam1* favours sparser models - links between nodes are increasingly more likely to be pruned). We consider independent gamma priors on the associations concerning confounding effects (*gam2*) in order to attenuate the strong effect of confounder and to artificially boost the importance of the link between 25-OHD and colorectal cancer. Overall, optimal models are the denser ones (characterised by smaller values of *gam1* parameter, most links remain in the model), and large positive DIC differences provide overwhelming evidence for a direct causal relation between low 25-OHD and colorectal cancer.

When focusing on denser models (*gam1*≤0.25), in 15/18 iterations there was overwhelming (DIC differences in the range of 10.6 to 3,919) and in 2 substantial (DIC differences of 9.7 and 5.2) evidence in favour of the full causal model, and in one iteration it was not possible to distinguish a preferred model with certainty, although the causal model was favoured (DIC difference = 3.2 units) (**Figure 3**). Results of all comparisons are shown in **Table 4** and more detail in **Table S1.**

**Table 4:** Likelihood of causal association between low 25-OHD and colorectal cancer (M7) is compared with the reverse causal hypothesis (proposing CRC leads to lower 25-OHD, M8), in a subset of data comprising a random sample of 500 cases and 500 controls.

		gam2												
		0.1		0.5		1.0		3.0		10.0		20.0		
gam1	MODEL	DIC	difference	DIC	difference		difference	DIC	difference	DIC	difference	DIC	difference	mean DIC
**0.025**	causal	−4415.1		−1663.6		−5545.9		−3414.2		−3707.8		−5481.7		
	reverse	−1563.2		−1565.4		−1564.4		−1564.3		−1565.4		−1562.4		
			2851.9		98.2		3981.5		1849.9		2142.4		3919.3	−2801.1
**0.1**	causal	−1884.7		−3511.6		−1727.2		−1944.3		−1773.4		−1568.8		
	reverse	−1567.3		−1560.6		−1574.4		−1565.2		−1561.8		−1559.1		
			317.4		1951.0		152.8		379.1		211.6		9.7	−1816.5
**0.25**	causal	−1569.3		−1572.8		−1892.8		−1565.6		−1623.8		−1579.4		
	reverse	−1564.1		−1562.2		−1565.1		−1562.4		−1563.3		−1562.3		
			5.2		10.6		327.7		3.2		60.5		17.1	−1598.6
**1**	causal	−1568.4		−1569.5		−1582.2		−1568.3		−1565.6		−1564.1		
	reverse	−1591.7		−1580		−1566.3		−1574.2		−1563.9		−1561.9		
			−23.3		−10.5		15.9		−5.9		1.7		2.2	−1571.3
**10**	causal	−1556.4		−1558.8		−1557.7		−1558.4		−1554.7		−1555.5		
	reverse	−1559.0		−1564.9		−1557.7		−1564.2		−1554.1		−1548.4		
			−2.6		−6.1		−0.1		−5.8		0.6		7.1	−1557.5

Deviance information criterion (DIC) score differences between two models are shown for a range of parameter settings; positive values indicate preference for the causal model. Mean DIC represents the average DIC including all causal and reverse causal models considered (lower mean DIC scores suggest better models), for any given setting of sparsity gam1 parameter (higher gam1 favours sparser models - links between nodes are increasingly more likely to be pruned). We consider independent gamma priors on the associations concerning confounding effects (gam2) in order to attenuate the strong effect of confounder and to artificially boost the importance of the link between 25-OHD and colorectal cancer. Overall, optimal models are the denser ones (characterised by smaller values of gam1 parameter, most links remain in the model), and large positive DIC differences provide overwhelming evidence for a direct causal relation between low 25-OHD and colorectal cancer. Details on DIC components are in **Table S1.**

* Noise parameters are set to: *precxt* = 1000, *precx* = 1000, *precy* = 0.1.

**Figure 4:**
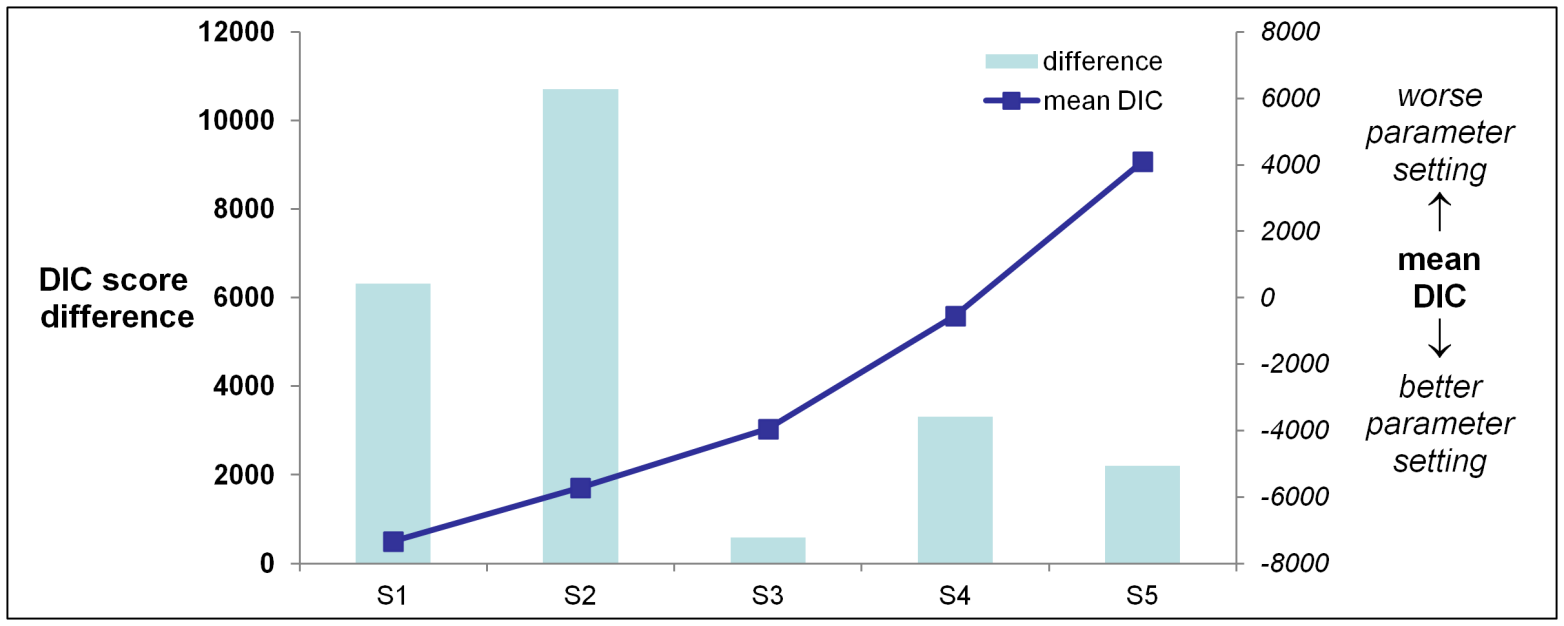
Likelihood of causal association between low 25-OHD and colorectal cancer is compared with the reverse causal hypothesis, (proposing CRC leads to lower 25-OHD), on the complete dataset and for a range of parameter settings. DIC score differences between models are shown; positive values indicate that causal association is more likely. Mean DIC (red line) is calculated as the average DIC for all causal and reverse causal models considered for any given parameter setting (smaller values indicate better models). Large positive DIC differences provide overwhelming evidence for a direct causal relation between low 25-OHD and colorectal cancer. * Settings: S1: precx = 1000, precxt = 1000, precy = 0.1; S2: precx = 100, precxt = 100, precy = 100; S3: precx = 1000, precxt = 1000, precy = 10; S4: precx = 100, precxt = 100, precy = 200; S5: precx = 20, precxt = 20, precy = 200.

Finally, we used all available data to compare full causal and full reverse causal models. We consistently observed evidence in support of the direct causal relation between low 25-OHD and CRC. Across all the noise parameter settings that we explored, *the full causal model provided a better explanation of data than the full reverse causal model*: DIC differences were between 580 and 10,715 units in favour of the full causal model (**Figure 4** and **Table 5** **, for DIC components see Table S2**).

**Table 5:** Likelihood of causal association between low 25-OHD and colorectal cancer is compared with the reverse causal hypothesis (proposing CRC leads to lower 25-OHD), on the complete dataset and for a range of parameter settings.

	setting				
	S1	S2	S3	S4	S5
**DIC causal**	−10,480	−11,074	−4,242	−2,203	3,004
**DIC reverse**	−4,161	−358	−3,661	1,115	5,214
**DIC difference**	6,319	10,715	580	3,318	2,210
**mean DIC**	−7,321	−5,716	−3,952	−544	4,109

Deviance information criterion (DIC) score differences between models are shown; positive values indicate that causal association is more likely. Mean DIC is calculated as the average DIC for all causal and reverse causal models considered for any given parameter setting (smaller values indicate better models). Large positive DIC differences provide overwhelming evidence for a direct causal relation between low 25-OHD and colorectal cancer. Details on DIC components are in **Table S2.** Digits after decimal point have been omitted from the table.

* Settings: S1: precx = 1000, precxt = 1000, precy = 0.1; S2: precx = 100, precxt = 100, precy = 100; S3: precx = 1000, precxt = 1000, precy = 10; S4: precx = 100,precxt = 100, precy = 200; S5: precx = 20, precxt = 20, precy = 200.

DIC scores computed here [51] generalize AIC scores used for inferring the direction of causality in LCMS [26], [53]. However, it has been argued that they may underpenalize model complexity [50]. By assuming that the full reverse model has approximately the same complexity as the full causal model, we additionally compared the best of the causal and reverse models according to their mean posterior deviances (Dbar). (Note that −1/2 Dbar may also be viewed as the “cooling limit” of thermodynamic integration used for approximating marginal likelihoods of the models [54]). Again, we found evidence of 11.5 natural log units in favour of the causal model.

Note that this is opposite to the results found by explicitly excluding the presence of hidden confounding (experiments 1 and 2); however, we have shown that according to the DIC scores, the models allowing for hidden confounders resulted in better explanations of the data than the models that did not allow for confounders. We also consistently observed that lower levels of 25-OHD are associated with CRC case status. Together, these results suggest that low plasma 25-OHD levels may be causally associated with CRC risk.

## Discussion

In this paper, we show evidence in support of a causal relationship between low plasma 25-OHD and colorectal cancer risk. The study was conducted by implementing novel methodology that extends the conventional instrumental variable approach and the more recent, likelihood-based causality model selection method [26], by accounting both for confounding by unknown factors and allowing pleiotropic relationships.

### SPIV and Improvement in the Methodology

Conventional approaches to the problem of causal inference are based on strong and often unrealistic assumptions about data. In practice such assumptions may be violated, which can lead to poor models and biased causal estimates [22], [55]. If one carefully selects instruments or sub-samples data to approximately satisfy the restrictive assumptions, inference in MR and LCMS is mathematically sound, but the results will generally be sensitive to the selections and can lead to varying conclusions [21], [46], [56]. In this paper we apply a different, model selection based strategy called SPIV, where we jointly consider genotypic factors predictive of either biomarkers or outcomes without relying on strong assumptions of the classical methods. The fact that the same “full causal” model explains the data better than alternative modelling hypotheses as shown for a broad range of domain-supported prior distributions is indicative of possible causality and justifies further controlled experiments.

The model selection based strategy underlying SPIV was advocated by some of the most prominent machine learning scientists [41], applied by Schadt et al. for a subset of models [26], further developed by Agakov et al. [46], and recently theoretically investigated by Winn [57]. It offers important extensions of the common methodology and may be used even in situations where relationships are pleiotropic or confounded by unknown/unmeasured factors (see **Table 6** and **Appendix S1** for more detail). Our approach can accommodate models underlying the conventional methods as limiting special cases.

**Table 6:** Comparison of the Sparse Instrumental Variable approach (SPIV) with Likelihood-based Causality Model Selection (LCMS) and Mendelian Randomization (MR).

	SPIV	LCMS	MR
**Pleiotropy**	yes	yes	no
**Latent confounding**	yes	no	yes
**Observation noise**	yes^1^	no	no
**Model selection**	yes	yes	no^2^
**Weak instruments** 3	yes	no	no

1 By observation noise we mean variations between true and observed biomarkers, and their different treatment in the underlying models.

2 Model selection implies probabilistic model comparison based on likelihood scores that can be used, for example, to infer the direction of causality. Note that while the classic MR can be used to compute p-values for causal and reverse models Timpson et al. (2011), it cannot be easily used to assess relative value of causal *vs* reverse causal explanations. In classic MR, formal and fair comparisons are further complicated by the fact that the causal and reverse models are not nested and use non-overlapping sets of instruments. The more recent Bayesian treatment of MR suggested by McKeigue et al. (2010) can in principle be used for model selection, but is limited to selecting either the conventional causal or non-causal explanation under the assumption of no pleiotropy.

3 Because SPIV is Bayesian and can use prior information to break symmetries between causal and reverse models, it can be used to infer the direction of causality even if only weak instruments are available.

We have previously described an inverse association between plasma 25-OHD concentration and CRC in this study population. However, results of Mendelian randomisation study we conducted were inconclusive [27].

In this study, by applying SPIV we consistently observed evidence in support of the direct causal relation between low 25-OHD and an increased risk of CRC, when pleiotropic and confounding effects were modelled explicitly, which is in agreement with previous work [58], [59]. Such inference became possible by relaxing the strong assumptions of common approaches and exploiting Bayesian model selection. Our results were consistent for a wide range of biologically plausible zero-centred heavy-tailed prior distributions.

We also show that models ignoring hidden confounders or pleiotropy have significantly worse likelihood-based scores then models accounting for them. This raises the question of reliability of causal inference in weaker models that ignore confounding and/or pleiotropy. Experiment 2 showed that the causal and reverse models considered by LCMS [26], [53] are inferior to models allowing for latent confounding. This also shows that Bayesian treatment of classic MR [44] would not be able to infer causality and would favour a non-causal explanation through confounders.

### Limitations and Future Work

An important limitation of our study is that only a small number of genotypes and environmental covariates were used as instruments, while there is overwhelming evidence that complex traits may potentially be explained by a very large number of common SNPs [60]. Future studies should consider employing larger number of genetic markers as instruments. Note that while high-dimensional instruments may be integrated into our framework relatively easily, this is less easy in the classic non-Bayesian methods due to the problems with weak instruments and possible overfitting [23], [24], [26], [53].

Another limitation of our strategy is that, in contrast to the standard approaches to causality, it does not formally guarantee equivalence of the causal “do-calculus” to probabilistic inference. However, our approach makes more realistic assumptions about the data and results in stronger models of the underlying phenomena, which is manifested by significantly better likelihood-based scores than models underlying the standard methods. In situations when the underlying models generating the data were known, the model selection approach similar to the one presented here has shown excellent quality of reconstruction of the underlying relationships (e.g. [46]; see also [26], [53] for earlier studies excluding latent variables where the biomarkers were gene expressions, and [57] for a recent theoretic formalization of the model selection approach to causal inference).

In the proposed Bayesian approach, inference is affected by prior distributions. This was partially mitigated by considering multiple such priors within the considered super-Gaussian family encoding sparsity in the posterior modes and allowing for rare large effects. In the future, other priors may be considered and effects investigated for a broader range of different assumptions about hyperparameters.

In this paper we have selected the best model by using the DIC criterion that balances predictive accuracy and model complexity. The DIC generalizes the Akaike Information Criterion [51], is easily available for MCMC samples, and often used in epidemiology [61]. The DIC scores consistently indicate preference for a causal hypothesis across the range of the considered priors. Also, for most of the considered settings, the DIC has resulted in a positive complexity estimate (pD, see **Table S1**). However, for other settings, the DIC may not be an accurate score, which may be the case e.g. for multi-modal posteriors [50], [62]. Other selection scores, e.g. the ones based on annealed importance sampling [63] and thermodynamic approximations of Bayes factors [54], [64] developed in statistical physics should be considered in the future, although they are significantly more expensive to compute. One technical limitation is the computational cost of the MCMC approximation of the Bayesian inference, which may be addressed in the future by considering alternative approaches to approximate inference [65].

Finally, it is currently unclear if colorectal cancer progression (or treatment) affects 25-OHD concentration. Our data has been collected at a single time point after the diagnosis. The apparent causal relationship suggested by this work may be further validated by collecting multiple temporally repeated measurements and replicating the analysis.

### Conclusion

Extended instrumental variable analysis (SPIV) indicates a causal association between low plasma 25-OHD and colorectal cancer risk. Our findings support the need for randomised clinical trials aimed at a further assessment of the role of vitamin D in colorectal cancer risk and suggest that investment in this field may be justified. With rising interest in instrumental variable approaches and Mendelian randomisation, it is important to be aware of the method limitations and requirements; failure to do so may seriously bias the inference of causality.

## Supporting Information

Figure S1 Examples of **A.** dense, **B.** sparse and **C.** decoupled models. The effect of increasing values of gamma sparsity parameter and consequential disappearing of the links in the model finally results in the decoupled model when gamma is large (eg. 10).
(TIF)

Table S1 Likelihood of causal association between low 25-OHD and colorectal cancer (M7) is compared with the reverse causal hypothesis (proposing CRC leads to lower 25-OHD, M8), in a subset of data comprising a random sample of 500 cases and 500 controls. DIC score differences between two models are shown for a range of parameter settings; positive values indicate preference for the causal model. Mean DIC represents the average DIC including all causal and reverse causal models considered (lower mean DIC scores suggest better models), for any given setting of sparsity gam1 parameter (higher gam1 favours sparser models - links between nodes are increasingly more likely to be pruned). We consider independent gamma priors on the associations concerning confounding effects (gam2) in order to attenuate the strong effect of confounder and to artificially boost the importance of the link between 25-OHD and colorectal cancer. Overall, optimal models are the denser ones (characterised by smaller values of gam1 parameter, most links remain in the model), and large positive DIC differences provide overwhelming evidence for a direct causal relation between low 25-OHD and colorectal cancer.
(DOC)

Table S2 Likelihood of causal association between low 25-OHD and colorectal cancer is compared with the reverse causal hypothesis (proposing CRC leads to lower 25-OHD), on the complete dataset and for a range of parameter settings. DIC components for both models are shown. Mean DIC is calculated as the average DIC for all causal and reverse causal models considered for any given parameter setting (smaller values indicate better models). Large positive DIC differences provide overwhelming evidence for a direct causal relation between low 25-OHD and colorectal cancer.
(DOC)

Methods S1 (DOCX)

Appendix S1 **On causality and improvement over conventional methods.**
(DOCX)
