# Supplementary material for: Model Selection Approach Suggests Causal Association between 25-Hydroxyvitamin D and Colorectal Cancer
Source: PLoS One. 2013 May 24;8(5):e63475. doi: 10.1371/journal.pone.0063475 (PMC3663843; doi:10.1371/journal.pone.0063475)
Supplement: Table S1 — Likelihood of causal association between low 25-OHD and colorectal cancer (M7) is compared with the reverse causal hypothesis (proposing CRC leads to lower 25-OHD, M8), in a subset of data comprising a random sample of 500 cases and 500 controls. DIC score differences between two models are shown for a range of parameter settings; positive values indicate preference for the causal model. Mean DIC represents the average DIC including all causal and reverse causal models considered (lower mean DIC scores suggest better models), for any given setting of sparsity gam1 parameter (higher gam1 favours sparser models - links between nodes are increasingly more likely to be pruned). We consider independent gamma priors on the associations concerning confounding effects (gam2) in order to attenuate the strong effect of confounder and to artificially boost the importance of the link between 25-OHD and colorectal cancer. Overall, optimal models are the denser ones (characterised by smaller values of gam1 parameter, most links remain in the model), and large positive DIC differences provide overwhelming evidence for a direct causal relation between low 25-OHD and colorectal cancer. (DOC) [file pone.0063475.s002.doc]

**Supplementary Table S1.** Likelihood of causal association between low 25-OHD and colorectal cancer (M7) is compared with the reverse causal hypothesis (proposing CRC leads to lower 25-OHD, M8), in a subset of data comprising a random sample of 500 cases and 500 controls. DIC score differences between two models are shown for a range of parameter settings; positive values indicate preference for the causal model. Mean DIC represents the average DIC including all causal and reverse causal models considered (lower mean DIC scores suggest better models), for any given setting of sparsity gam1 parameter (higher gam1 favours sparser models - links between nodes are increasingly more likely to be pruned). We consider independent gamma priors on the associations concerning confounding effects (gam2) in order to attenuate the strong effect of confounder and to artificially boost the importance of the link between 25-OHD and colorectal cancer. Overall, optimal models are the denser ones (characterised by smaller values of gam1 parameter, most links remain in the model), and large positive DIC differences provide overwhelming evidence for a direct causal relation between low 25-OHD and colorectal cancer.

| **gam2** | **model** | **gam1=0.025** | | | |  | **gam1=0.1** | | | |  | **gam1=0.25** | | | |  | **gam1=1** | | | |  | **gam1=10** | | | |
| --- | --- | --- | --- | --- | --- | --- | --- | --- | --- | --- | --- | --- | --- | --- | --- | --- | --- | --- | --- | --- | --- | --- | --- | --- | --- |
|  |  |  |  |
| **0.1** |  | Dbar | Dhat | pD | DIC |  | Dbar | Dhat | pD | DIC |  | Dbar | Dhat | pD | DIC |  | Dbar | Dhat | pD | DIC |  | Dbar | Dhat | pD | DIC |
|  | **causal** | -2925.4 | -1435.6 | -1489.8 | -4415.1 |  | -2922.4 | -3960.1 | 1037.7 | -1884.7 |  | -2922.7 | -4276.1 | 1353.4 | -1569.3 |  | -2922.8 | -4277.2 | 1354.4 | -1568.4 |  | -2916.9 | -4277.3 | 1360.5 | -1556.4 |
|  | **reverse** | -2919.53 | -4275.86 | 1356.3 | -1563.2 |  | -2921.4 | -4275.4 | 1354.1 | -1567.3 |  | -2920.1 | -4276.2 | 1356.1 | -1564.1 |  | -2928.6 | -4265.5 | 1336.9 | -1591.7 |  | -2916.6 | -4274.2 | 1357.6 | -1559.0 |
| **0.5** |  |  |  |  |  |  |  |  |  |  |  |  |  |  |  |  |  |  |  |  |  |  |  |  |  |
|  | **causal** | -2917.6 | -4171.6 | 1254.0 | -1663.6 |  | -2920.1 | -2328.6 | -591.5 | -3511.6 |  | -2922.0 | -4271.1 | 1349.2 | -1572.8 |  | -2922.4 | -4275.2 | 1352.8 | -1569.5 |  | -2915.6 | -4277.1 | 1361.4 | -1554.2 |
|  | **reverse** | -2919.7 | -4273.9 | 1354.3 | -1565.4 |  | -2917.8 | -4274.9 | 1357.2 | -1560.6 |  | -2919.1 | -4276.0 | 1356.9 | -1562.2 |  | -2925.7 | -4271.4 | 1345.7 | -1580.0 |  | -2917.9 | -4270.9 | 1353.0 | -1564.9 |
| **1** |  |  |  |  |  |  |  |  |  |  |  |  |  |  |  |  |  |  |  |  |  |  |  |  |  |
|  | **causal** | -2926.5 | -307.0 | -2619.5 | -5545.9 |  | -2917.7 | -4108.2 | 1190.5 | -1727.2 |  | -2920.1 | -3947.3 | 1027.3 | -1892.8 |  | -2921.1 | -4260.0 | 1338.9 | -1582.2 |  | -2915.5 | -4277.4 | 1361.9 | -1553.6 |
|  | **reverse** | -2919.8 | -4275.1 | 1355.4 | -1564.4 |  | -2925.0 | -4275.7 | 1350.7 | -1574.4 |  | -2921.3 | -4277.5 | 1356.2 | -1565.1 |  | -2921.7 | -4277.2 | 1355.5 | -1566.3 |  | -2916.4 | -4275.1 | 1358.7 | -1557.7 |
| **3** |  |  |  |  |  |  |  |  |  |  |  |  |  |  |  |  |  |  |  |  |  |  |  |  |  |
|  | **causal** | -2926.3 | -2438.5 | -487.9 | -3414.2 |  | -2923.1 | -3901.9 | 978.8 | -1944.3 |  | -2919.8 | -4274.1 | 1354.2 | -1565.6 |  | -2922.3 | -4276.2 | 1354.0 | -1568.3 |  | -2917.9 | -4277.4 | 1359.5 | -1558.4 |
|  | **reverse** | -2920.5 | -4276.6 | 1356.1 | -1564.3 |  | -2920.0 | -4274.8 | 1354.8 | -1565.2 |  | -2919.1 | -4275.8 | 1356.7 | -1562.4 |  | -2922.7 | -4271.2 | 1348.5 | -1574.2 |  | -2919.4 | -4274.6 | 1355.2 | -1564.2 |
| **10** |  |  |  |  |  |  |  |  |  |  |  |  |  |  |  |  |  |  |  |  |  |  |  |  |  |
|  | **causal** | -2920.6 | -2133.3 | -787.3 | -3707.8 |  | -2923.4 | -4073.4 | 1150.0 | -1773.4 |  | -2922.2 | -4220.6 | 1298.4 | -1623.8 |  | -2921.0 | -4276.4 | 1355.4 | -1565.6 |  | -2915.9 | -4277.1 | 1361.2 | -1554.7 |
|  | **reverse** | -2920.3 | -4275.2 | 1354.9 | -1565.4 |  | -2919.0 | -4276.2 | 1357.2 | -1561.8 |  | -2919.7 | -4276.1 | 1356.4 | -1563.3 |  | -2920.2 | -4276.5 | 1356.3 | -1563.9 |  | -2915.3 | -4276.5 | 1361.2 | -1554.1 |
| **20** |  |  |  |  |  |  |  |  |  |  |  |  |  |  |  |  |  |  |  |  |  |  |  |  |  |
|  | **causal** | -2926.2 | -370.7 | -2555.5 | -5481.7 |  | -2920.8 | -4272.9 | 1352.1 | -1568.8 |  | -2925.2 | -4270.9 | 1345.8 | -1579.4 |  | -2920.7 | -4277.3 | 1356.6 | -1564.1 |  | -2916.5 | -4277.6 | 1361.0 | -1555.5 |
|  | **reverse** | -2919.3 | -4276.2 | 1356.9 | -1562.4 |  | -2917.3 | -4275.4 | 1358.1 | -1559.1 |  | -2919.0 | -4275.6 | 1356.7 | -1562.3 |  | -2918.6 | -4275.3 | 1356.7 | -1561.9 |  | -2912.9 | -4277.4 | 1364.5 | -1548.4 |
