# Supplementary material for: Model Selection Approach Suggests Causal Association between 25-Hydroxyvitamin D and Colorectal Cancer
Source: PLoS One. 2013 May 24;8(5):e63475. doi: 10.1371/journal.pone.0063475 (PMC3663843; doi:10.1371/journal.pone.0063475)
Supplement: Table S2 — Likelihood of causal association between low 25-OHD and colorectal cancer is compared with the reverse causal hypothesis (proposing CRC leads to lower 25-OHD), on the complete dataset and for a range of parameter settings. DIC components for both models are shown. Mean DIC is calculated as the average DIC for all causal and reverse causal models considered for any given parameter setting (smaller values indicate better models). Large positive DIC differences provide overwhelming evidence for a direct causal relation between low 25-OHD and colorectal cancer. (DOC) [file pone.0063475.s003.doc]

**Supplementary Table S2.** Likelihood of causal association between low 25-OHD and colorectal cancer is compared with the reverse causal hypothesis (proposing CRC leads to lower 25-OHD), on the complete dataset and for a range of parameter settings. DIC components for both models are shown. Mean DIC is calculated as the average DIC for all causal and reverse causal models considered for any given parameter setting (smaller values indicate better models). Large positive DIC differences provide overwhelming evidence for a direct causal relation between low 25-OHD and colorectal cancer.

| **Setting/ model** | **Dbar** | **Dhat** | **pD** | **DIC** | **DIC difference** | **mean DIC** |
| --- | --- | --- | --- | --- | --- | --- |
| **setting 1** |  |  |  |  |  |  |
| full causal | -7678.32 | -4876.54 | -2801.78 | -10480.1 |  |  |
| full reverse | -7655.07 | -11148.8 | 3493.78 | -4161.29 |  |  |
|  |  |  |  |  | 6318.81 | -7320.7 |
| **setting 2** |  |  |  |  |  |  |
| full causal | 482.384 | -2038.85 | 2521.24 | 3003.62 |  |  |
| full reverse | 2097.85 | -1018.04 | 3115.89 | 5213.74 |  |  |
|  |  |  |  |  | 10715.28 | -5716.06 |
| **setting 3** |  |  |  |  |  |  |
| full causal | -1098.49 | 8876.75 | -9975.24 | -11073.7 |  |  |
| full reverse | -1623.51 | -2888.61 | 1265.09 | -358.417 |  |  |
|  |  |  |  |  | 580.27 | -3951.62 |
| **setting 4** |  |  |  |  |  |  |
| full causal | -1585.34 | -968.138 | -617.201 | -2202.54 |  |  |
| full reverse | -1194.85 | -3505.11 | 2310.26 | 1115.41 |  |  |
|  |  |  |  |  | 3317.95 | -543.57 |
| **setting 5** |  |  |  |  |  |  |
| full causal | -6012.05 | -7782.35 | 1770.3 | -4241.75 |  |  |
| full reverse | -6023.56 | -8385.63 | 2362.08 | -3661.48 |  |  |
|  |  |  |  |  | 2210.12 | 4108.68 |
