# Supplementary material for: Model Selection Approach Suggests Causal Association between 25-Hydroxyvitamin D and Colorectal Cancer
Source: PLoS One. 2013 May 24;8(5):e63475. doi: 10.1371/journal.pone.0063475 (PMC3663843; doi:10.1371/journal.pone.0063475)
Supplement: Methods S1 — (DOCX) [file pone.0063475.s004.docx]

**Supporting Information**

**SUPPLEMENTARY METHODS S1**

**Genotyping.** Case and control DNA samples were stored, genotyped and analysed in the same way. 2,000 subjects (1,000 cases and 1,000 controls) were genotyped as part of an array-based candidate gene approach, using the Illumina Infinium I Custom array platform and performed by Illumina (San Diego). For participants for which genome-wide scan was not available, genotyping was undertaken using TaqMan in the Wellcome Trust Clinical Research Facility (WTCRF) in Edinburgh. Data were subject to Illumina or WTCRF quality control procedures. Assumptions of Hardy-Weinberg Equilibrium (HWE) were tested using a chi-squared test.

**Basic model.** The basic model and its components are shown in **Figure 1**. This is in accordance with the Sparse Instrumental Variable approach (SPIV) introduced recently by Agakov et al. [[45](#_ENREF_45),[46](#_ENREF_46),[47](#_ENREF_47)]. Noteworthy extensions to conventional Mendelian Randomisation and instrumental variable approaches comprise: presence of pleiotropic links, presence of “unmeasured confounders” (ie. unmeasured variables that can affect biomarker and/or disease) and accounting for the noise in the measurement.

A range of different priors on parameters of the linear mappings could be considered. Here we explored the double-exponential (Laplace) construction of [[49](#_ENREF_49)], used e.g. by LASSO and related methods. This favours sparsity at the mode of the posterior distribution of the parameters given the data, and allows for the possibility of rare large effects due to the heavy tails of the Laplacian. Our prior biological knowledge supports retaining all of the links, as only variables previously associated to 25-OHD or CRC were included in the model in the first place. However, we also tested sparser models (with the concentration parameter gamma ≥1), expecting experiments to become largely uninformative as sparsity increases and models become approximately decoupled, in which case any difference between causal and reverse causal model is likely to be due to the noise. This was indeed confirmed in experiments.

To account for measurement error, we introduce precision terms: *precx*, *precxt*, *precy* and *precz*; these are precisions (inverse variances) of noise terms to be associated with biomarker, measurement of biomarker, disease status and unmeasured confounders, respectively. Smaller values are associated with wider confidence intervals associated with every measurement. We repeat analysis for a range of different gamma and noise parameter settings.

To parameterize the model, we have largely followed the construction of [[46](#_ENREF_46)], but assumed a binary outcome variable y (CRC status) and a sparse logistic regression model for the probability of the outcome given the genotypes, biomarker, and latent confounder. However, in our case due to the well-defined sets of biomarkers and instruments, we were able to apply the full Bayesian approach to model comparison using Markov Chain Monte Carlo. To compare different models, primarily causal and reverse causal models (represented by the direction of the link *w* in **Figure 1**) we have set up the SPIV model using WinBUGS statistical software for Bayesian analysis using Markov chain Monte Carlo methods [[52](#_ENREF_52)].

**Missing data**. Initial cohort comprised of 5,669 individuals for whom plasma 25-OHD and genetic data were available. Although WinBUGS allows for missing data in the predictor variables by specifying priors on the missing predictors, we thought that modelling such observations directly when they are only used for the study of the causal relation between the biomarker and outcome would be a waste of modelling effort; also any inference of the missing predictors would be very computationally expensive. Therefore, after exclusions, 2645 individuals were included in the analysis (1057 cases and 1588 controls).

**Sparsity parameter.** Gamma (*gam1* and *gam2*) are concentration parameters of the L1 (Laplace) priors on the model parameters. A higher gamma drives sparsity in the posterior mode of the models' parameters. When the posterior mode is used in order to estimate importance of a predictor variable, such models are more likely to have their links seen as unimportant and pruned, in which case models with fewer links are favoured. In our experiments we repeat analysis for a range of different gamma settings.

**Noise parameters.** Measurements are never perfectly accurate, firstly because of biological variability (eg. 25-OHD changes with seasons) and secondly because of the inevitable measurement error. For this reason, we introduce precision terms: *precx*, *precxt*, *precy* and *precz*; these are precisions (inverse variances) of noise terms to be associated with biomarker, measurement of biomarker, disease status and unmeasured confounders, respectively. The noise in measurement is calculated as:

±√(1/prec) * 3, for confidence interval (CI) of 99%.

For example, the *precxt* = 200 means that, with 99% certainty, a true measurement of biomarker lies within ±0.21 of the measured (and scaled) value. Smaller values (eg. *precxt*=100) are associated with wider confidence intervals, while larger values effectively suggest we trust measurements more, ie. confidence intervals associated with every measurement are smaller. Had multiple repeated measurements been available, we would be able to set the precision terms with more certainty. In our experiments, we considered two approaches to handling the observation noise. First, we ran the experiments with several fixed noise models. We then considered setting Gamma priors on the precisions rather than fixing them at specified values, which empirically did not significantly affect inference of the causal direction.

**Unmeasured, hidden confounders.** Unmeasured confounders represent missing, unmeasured factors that affect biomarker and/or the outcome. By definition, we do not have prior knowledge of unmeasured confounders so they need to be modelled. We marginalise out the unmeasured confounders, assuming they have Gaussian prior with mean=0 and unit variance, to get the complete model that is most likely to generate our observed data. Unmeasured confounders are coupled with a noise term *precz*. *Precz* is set to 1, to be on the same scale as observed data (data was scaled prior to analysis, as described above).

**Relation between noise and unmeasured confounders.** Unsurprisingly, we observe an inverse relationship between the importance of accounting for unmeasured confounders and permitted levels of noise in the data. This is because assuming higher levels of noise in the data indirectly accounts for some confounding.

**Comparison of models.** To determine model convergence, the Gelman-Rubin convergence statistic was monitored. The comparison of models was achieved by looking at their total DIC scores. DIC is the “Deviance Information Criterion”, which generalizes other criteria used for model comparison. It is calculated by WinBUGS programme and given by:

DIC = Dbar + pD = Dhat + 2 pD.

The model with the smallest DIC was estimated to be the model that would best predict a replicate dataset, ie. it is the model that fits the data best. Very roughly, differences in DIC of more than 10 would definitely rule out the model with the higher DIC and differences between 5 and 10 are substantial [[52](#_ENREF_52)]. Differences below 5 are more difficult to interpret, but if consistent over different model settings, suggest that model with lower DIC is the more likely one. We also report mean DIC score across all investigated parameter settings, to assess which parameter setting is best.

To calculate approximately how much more likely one model is compared to the other, we exponentiate the difference in DIC scores of the two models:

Exp (abs (DIC_M1_ – DIC_M2_) ).

For example, DIC of model 1 = 352.5 and of model 2 = 349.5, gives us a DIC difference of 3, and suggests that model 2 is approximately 20 times more likely than model 1 (exp(3)=20.09).
