# Supplementary material for: Model Selection Approach Suggests Causal Association between 25-Hydroxyvitamin D and Colorectal Cancer
Source: PLoS One. 2013 May 24;8(5):e63475. doi: 10.1371/journal.pone.0063475 (PMC3663843; doi:10.1371/journal.pone.0063475)
Supplement: Appendix S1 — On causality and improvement over conventional methods. (DOCX) [file pone.0063475.s005.docx]

**Supporting Information**

**APPENDIX S1**

**Causality**

Whether or not causation may be inferred from observational data has been a matter of philosophical debate. [[42](#_ENREF_42),[43](#_ENREF_43)] argues that causal assumptions cannot be verified unless one makes a recourse to experimental control, and that there is nothing in the probability distribution p(x, y) which can tell whether a change in x may have an effect on y. Traditional discussions of causality are largely focused on determining sets of graph-theoretic *conditions* when a post-intervention distribution p(y|do(x)) may be uniquely determined from a pre-intervention distribution p(y, x, z) (see e.g. references in [[46](#_ENREF_46)]). If the causal effects are shown to be identifiable, their magnitudes can be obtained by statistical estimation, which for common models often reduces to solving systems of linear equations. In contrast, from the Bayesian perspective, the causality detection problem may be viewed as that of model selection, where a model x→y is compared with y→x. The problem is complicated by the likelihood-equivalence, where for each setting of parameters of one model there may exist a setting of parameters of the other giving rise to the identical likelihoods. However, unless the priors are chosen in such a way that Mx→y and My→x also have identical posteriors, it may be possible to infer the direction of the arrow – in particular, the fact that the same model is preferred for a range of priors reflecting the domain knowledge, may indicate the direction of causality and help to focus future controlled experiments. The view that the priors of likelihood-equivalent models do not need to be set to ensure the equivalence was defended e.g. by [[41](#_ENREF_41)]. This work is based on the model selection based approach to learning structures of latent variable models, where the atomic generic model shown on **Figure 1** is inspired by the probabilistic graphical modelling view of instrumental variable analysis, extended for pleiotropy, noise, and latent variables. Specific modelling hypotheses are tested by comparing deviance information criterion scores readily computable from MCMC samples for the corresponding direct, reverse, and pleiotropic models with and without latent confounders. The approach is largely motivated by the observation that independent variables do not establish a causal relation, while strong unconfounded direct dependencies retained in the posterior modes even under large sparseness-inducing penalties may indicate potential causality and suggest candidate markers for further controlled experiments or studies with temporally repeated measurements.

**Improvement over Conventional Methods**

Advantages of SPIV over more conventional Mendelian Randomization and Likelihood-based Causality Model Selection approaches are summarized in **Table 6**. To clarify this further, we note that:

(1) In order for inferences from conventional MR analysis to be reliable, the assumption of no pleiotropy is required [[20](#_ENREF_20)]. The validity of this assumption cannot be generally guaranteed [[66](#_ENREF_66)]. Examples that challenge it in our study are: (i) vitamin D receptor gene (VDR) and CDH1 (gene associated with CRC) are co-expressed and (ii) vitamin D response elements are present in multiple genes associated with CRC [[67](#_ENREF_67),[68](#_ENREF_68)]. In contrast to MR and classic instrumental variable methods, our approach SPIV allows for pleiotropic links.

(2) One of the core problems of the conventional LCMS analysis [[26](#_ENREF_26),[53](#_ENREF_53)] is inability to handle latent confounding. It is particularly difficult to deal with confounding as some known factors cannot be measured and hidden confounding factors are likely to exist; therefore, in practice, one can never be sure that all relevant confounders have been measured and accounted for [[20](#_ENREF_20)]. While MR can in principle handle confounding under the assumption of no pleiotropy, it has been argues that instrumental variables can be less useful when strong confounding exists due to common violations of IV assumptions [[55](#_ENREF_55)]. By using SPIV, we account for unmeasured and hidden confounders by explicitly modelling them in the analysis as latent variables (random effects), and investigate consistency of the modelling hypotheses for different prior distributions on the strength of confounding effects.

(3) In contrast to LCMS and MR, our method allows for a different treatment of true and underlying biomarkers.

(4) In contrast to classic MR concerned with estimation of the magnitude of causal effects, SPIV is based on model selection and can be used to infer the direction of causality. Note that while the classic MR estimation can be used to compute p-values for causal and reverse models [[25](#_ENREF_25)], it cannot be easily applied to assess relative value of causal *vs.* reverse causal explanations. In classic MR, formal and fair comparisons are further complicated by the fact that the causal and reverse models are not nested and use non-overlapping sets of instruments. The more recent Bayesian treatment of MR suggested by [[44](#_ENREF_44)] can in principle be used for model selection, but is limited to selecting either the conventional causal or non-causal explanation under the assumption of no pleiotropy.

(5) SPIV allows easy inclusion of multiple covariates and is not dependent on the strength of the instruments. This helps to address the additional caveat of MR studies [[69](#_ENREF_69),[70](#_ENREF_70)] which are particularly challenging when multiple instruments are required [[23](#_ENREF_23),[24](#_ENREF_24)].

**Supplementary References.**

66. Sivakumaran S, Agakov F, Theodoratou E, Prendergast JG, Zgaga L, et al. (2011) Abundant pleiotropy in human complex diseases and traits. Am J Hum Genet 89: 607-618.

67. Palmer HG, Gonzalez-Sancho JM, Espada J, Berciano MT, Puig I, et al. (2001) Vitamin D(3) promotes the differentiation of colon carcinoma cells by the induction of E-cadherin and the inhibition of beta-catenin signaling. J Cell Biol 154: 369-387.

68. Ramagopalan SV, Heger A, Berlanga AJ, Maugeri NJ, Lincoln MR, et al. (2010) A ChIP-seq defined genome-wide map of vitamin D receptor binding: associations with disease and evolution. Genome Res 20: 1352-1360.

69. Burgess S, Thompson SG (2011) Bias in causal estimates from Mendelian randomization studies with weak instruments. Stat Med 30: 1312-1323.

70. Pierce BL, Ahsan H, Vanderweele TJ (2011) Power and instrument strength requirements for Mendelian randomization studies using multiple genetic variants. Int J Epidemiol 40: 740-752.
